# 1,2-Bis(*N*′-benzoyl­thio­ureido)benzene

**DOI:** 10.1107/S1600536808008374

**Published:** 2008-04-02

**Authors:** Elhadj Ibrahima Thiam, Mayoro Diop, Mohamed Gaye, Abdou Salam Sall, Aliou Hamady Barry

**Affiliations:** a Département de Chimie, Faculté des Sciences et Techniques, Université Cheikh Anta Diop, Dakar, Senegal; b Département de Chimie, Faculté des Sciences, Université de Nouakchott, Nouakchott, Mauritania

## Abstract

The title compound, C_22_H_18_N_4_O_2_S_2_, was characterized by ^1^H and ^13^C NMR, solid-state IR spectroscopy and X-ray crystallographic techniques. The crystal structure determination reveals that the twisting modes of the two side arms are different [C—N—C—O and C—N—C—N torsion angles = −1.2 (3) and 1.1 (3)°, respectively, in one arm and 24.1 (3) and −5.1 (3)°, respectively, in the other]. The crystal structure involves N—H⋯O and N—H⋯S hydrogen bonds.

## Related literature

For related structures: see Arslan *et al.* (2004 ▶); Avşar *et al.* (2003 ▶).
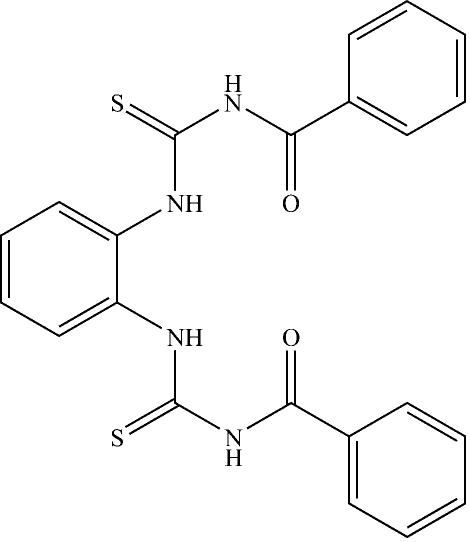


## Experimental

### 

#### Crystal data

C_22_H_18_N_4_O_2_S_2_
*M* *_r_* = 434.52
Triclinic, 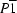

*a* = 7.179 (1) Å
*b* = 12.064 (2) Å
*c* = 12.476 (2) Å
α = 77.88 (5)°
β = 86.96 (5)°
γ = 77.91 (5)°
*V* = 1032.9 (3) Å^3^
*Z* = 2
Mo *K*α radiation
μ = 0.28 mm^−1^
*T* = 173 (2) K
0.10 × 0.10 × 0.10 mm

#### Data collection

Nonius KappaCCD diffractometer
Absorption correction: none
12922 measured reflections
4671 independent reflections
3234 reflections with *I* > 2σ(*I*)
*R*_int_ = 0.046

#### Refinement

*R*[*F* ^2^ > 2σ(*F* ^2^)] = 0.047
*wR*(*F* ^2^) = 0.118
*S* = 1.04
4671 reflections
271 parameters
H-atom parameters constrained
Δρ_max_ = 0.17 e Å^−3^
Δρ_min_ = −0.24 e Å^−3^

### 

Data collection: *COLLECT* (Nonius, 1998 ▶); cell refinement: *DENZO* (Nonius, 1998 ▶); data reduction: *DENZO*; program(s) used to solve structure: *SHELXS97* (Sheldrick, 2008 ▶); program(s) used to refine structure: *SHELXL97* (Sheldrick, 2008 ▶); molecular graphics: *PLATON* (Spek, 2003 ▶); software used to prepare material for publication: *SHELXL97*.

## Supplementary Material

Crystal structure: contains datablocks I, global. DOI: 10.1107/S1600536808008374/ww2115sup1.cif

Structure factors: contains datablocks I. DOI: 10.1107/S1600536808008374/ww2115Isup2.hkl

Additional supplementary materials:  crystallographic information; 3D view; checkCIF report

## Figures and Tables

**Table 1:** Hydrogen-bond geometry (Å, °)

*D*—H⋯*A*	*D*—H	H⋯*A*	*D*⋯*A*	*D*—H⋯*A*
N1—H*N*1⋯O6	0.88	1.88	2.633 (2)	142
N2—H*N*2⋯O7	0.88	1.99	2.680 (2)	134
N4—H*N*4⋯S2^i^	0.88	2.61	3.478 (2)	170

Symmetry code: (i) 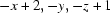.

**Fig. 1.**
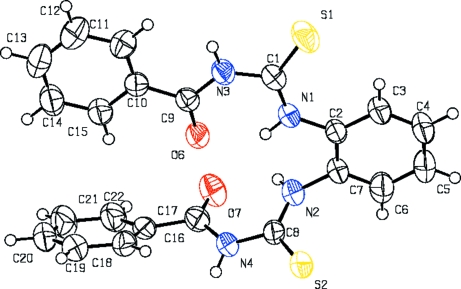
An ORTEP view of the asymmetric unit of the title compound, showing the atom-numbering scheme. Displacement ellipsoids are plotted at the 50% probability level.

**Fig. 2.**
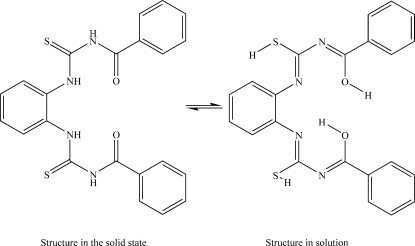
The tautomerism in the title compound.

## supplementary crystallographic information

### Comment

The title compound, C_22_H_18_N_4_O_2_S_2_, was characterized by ^1^H and ^13^C NMR, solid-state IR and X-ray crystallographic techniques. The X-ray structure determination reveals that the compound crystallizes in the triclinic space group P-1 with one molecule in the asymmetric unit. The molecular geometry is illustrated in Fig. 1. The S1—C1 [1.6574 (18)Å], the S2—C8 [1.660 (2)Å], the O7—C16 [1.219 (2)Å] and O6—C9 [1.222 (2)Å] distances indicates that these correspond to double bonds and are comparable to those observed for 1-(biphenyl-4-carbonyl)-3-*p*-tolyl-thiourea [1.647 (3)Å for C—S, 1.217 (3) and 1.224 (3)Å for C—O respectively (Arslan *et al.*, 2004)]. The C—N bond lengths are in the range [1.335 (2) - 1.435 (2)Å] and are shorter than the normal single C—N bond length, indicating double bond character (Avşar *et al.*, 2003). The two side arms are not twisted in the same way. One of the arms is slightly twisted as reflected by the two torsion angle C1—N3—C9—O6 [-1.2 (3)°] and C9—N3—C1—N1 [1.1 (3) °]. The torsion angles C8—N4—C16—O7 [24.1 (3)°] and C16—N4—C8—N2 [-5.1 (3)°] in the other side arm indicate that this one is more strongly twisted. Intramolecular hydrogen-bond contacts involve the NH groups and the O atoms of the amide groups as well as the N atoms of the thiourea groups while intermolecular hydrogen-bond is observed between the NH groups and the S atoms of the thiourea groups (Table 2)

### Experimental

All purchased chemicals and solvents were reagent grade and used without further purifcation. Melting points were determined with a Büchi 570 melting-point apparatus and were uncorrected. The ^1^H NMR spectra were measured with a Bruker AM-400 spectrometer, using tetramethylsilane as the internal standard. The solid-state IR spectra were recorded from KBr discs on a Nicolet spectrophotometer. To a mixture of 9.718 g (100 mmol) of potassium thiocyanate and 100 ml of acetone was added dropwise a solution of 14.071 g (100 mmol) of benzoyl chloride in 50 ml of acetone. The resulting mixture was stirred under reflux for 1 h and cooled to room temperature. A solution of 5.407 g (50 mmol) of 1,2-diaminobenzene in 20 ml of acetone was added. The yellow solution obtained was stirred at room temperature during 2 h. 300 ml of 1 N HCl was added to dissolve the precipitated KCl. A white solid appeared after five minutes. The compound was filtered and washed with 3 x 50 ml of water and dried under vacuum. Recrystallization from a mixture of methanol and chloroform (1:1) gave 18.52 g (85.7%) of the title compound. *M*.p. 360±362 K (uncorrected. Mass spectrum, m/*z*= 434 (*M*^+^). ^1^H NMR (CDCl_3_): δ 7.38–7.93 (m, 14H, ArH), 9.30 (s, 2H, SH), 12.36 (s, 2H, OH); ^13^C NMR (CDCl_3_): δ 126.97, 127.70, 128.15, 129.10, 131.14, 131.60, 133.10, 166.90, 180. IR (cm^-1^,KBr): 3210, 1673, 1596, 1470, 1319, 1262, 1149, 857, 688. Analysis calculated for C_22_H_18_N_4_O_2_S_2_: C 60.81, H 4.18, N 12.89, S 14.76%; found: C 60.80, H 4.59, N 12.87, S 14.80%. Monocrystals suitable for X-ray analysis was obtained from slow evaporation of a dimethylformamide solution of the product.

### Refinement

All H atoms were placed geometrically and refined with a riding model. *U*_iso_(H) for H was assigned as 1.2 *U*_eq_ of the attached C or N atoms.

### Crystal data

**Table:**

C_22_H_18_N_4_O_2_S_2_	*Z* = 2
*M**_r_* = 434.52	*F*_000_ = 452
Triclinic, *P*1	*D*_x_ = 1.397 Mg m^−^^3^
Hall symbol: -P 1	Mo *K*α radiation λ = 0.71069 Å
*a* = 7.179 (1) Å	Cell parameters from 6418 reflections
*b* = 12.064 (2) Å	θ = 1.0–27.5º
*c* = 12.476 (2) Å	µ = 0.29 mm^−^^1^
α = 77.88 (5)º	*T* = 173 (2) K
β = 86.96 (5)º	Prism, colorless
γ = 77.91 (5)º	0.10 × 0.10 × 0.10 mm
*V* = 1032.9 (3) Å^3^	

### Data collection

**Table:**

Nonius KappaCCD diffractometer	3234 reflections with *I* > 2σ(*I*)
Radiation source: fine-focus sealed tube	*R*_int_ = 0.047
Monochromator: graphite	θ_max_ = 27.4º
*T* = 173(2) K	θ_min_ = 3.1º
ω scans	*h* = −9→8
Absorption correction: none	*k* = −15→15
12922 measured reflections	*l* = −16→14
4671 independent reflections	

### Refinement

**Table:**

Refinement on *F*^2^	Secondary atom site location: difference Fourier map
Least-squares matrix: full	Hydrogen site location: inferred from neighbouring sites
*R*[*F*^2^ > 2σ(*F*^2^)] = 0.047	H-atom parameters constrained
*wR*(*F*^2^) = 0.118	*w* = 1/[σ^2^(*F*_o_^2^) + (0.048*P*)^2^ + 0.1332*P*] where *P* = (*F*_o_^2^ + 2*F*_c_^2^)/3
*S* = 1.04	(Δ/σ)_max_ < 0.001
4671 reflections	Δρ_max_ = 0.17 e Å^−^^3^
271 parameters	Δρ_min_ = −0.24 e Å^−^^3^
Primary atom site location: structure-invariant direct methods	Extinction correction: none

### Special details

**Table:**

Geometry. All e.s.d.'s (except the e.s.d. in the dihedral angle between two l.s. planes) are estimated using the full covariance matrix. The cell e.s.d.'s are taken into account individually in the estimation of e.s.d.'s in distances, angles and torsion angles; correlations between e.s.d.'s in cell parameters are only used when they are defined by crystal symmetry. An approximate (isotropic) treatment of cell e.s.d.'s is used for estimating e.s.d.'s involving l.s. planes.
Refinement. Refinement of *F*^2^ against ALL reflections. The weighted *R*-factor *wR* and goodness of fit *S* are based on *F*^2^, conventional *R*-factors *R* are based on *F*, with *F* set to zero for negative *F*^2^. The threshold expression of *F*^2^ > σ(*F*^2^) is used only for calculating *R*-factors(gt) *etc*. and is not relevant to the choice of reflections for refinement. *R*-factors based on *F*^2^ are statistically about twice as large as those based on *F*, and *R*- factors based on ALL data will be even larger.

### Fractional atomic coordinates and isotropic or equivalent isotropic displacement parameters (Å^2^)

**Table:**

	*x*	*y*	*z*	*U*_iso_*/*U*_eq_	
S1	0.51586 (8)	0.37259 (4)	0.93513 (5)	0.0727 (2)	
S2	0.83170 (8)	0.17089 (4)	0.50551 (4)	0.06579 (18)	
O6	0.69327 (18)	0.01181 (10)	0.86202 (10)	0.0574 (3)	
O7	0.5045 (2)	−0.09194 (11)	0.70005 (13)	0.0680 (4)	
N1	0.5437 (2)	0.23196 (11)	0.78892 (12)	0.0473 (3)	
HN1	0.5932	0.1610	0.7809	0.057*	
N2	0.5204 (2)	0.12967 (12)	0.61750 (12)	0.0529 (4)	
HN2	0.4517	0.0797	0.6504	0.064*	
N3	0.6479 (2)	0.15035 (12)	0.96355 (12)	0.0515 (4)	
HN3	0.6623	0.1608	1.0301	0.062*	
N4	0.7603 (2)	−0.02950 (12)	0.61097 (12)	0.0536 (4)	
HN4	0.8699	−0.0573	0.5818	0.064*	
C1	0.5672 (2)	0.25055 (15)	0.88870 (15)	0.0487 (4)	
C2	0.4547 (2)	0.30447 (14)	0.69408 (14)	0.0464 (4)	
C3	0.3759 (3)	0.42184 (16)	0.68239 (17)	0.0617 (5)	
H3	0.3854	0.4605	0.7402	0.074*	
C4	0.2842 (3)	0.48218 (17)	0.5872 (2)	0.0740 (6)	
H4	0.2306	0.5623	0.5801	0.089*	
C5	0.2688 (3)	0.4288 (2)	0.5025 (2)	0.0798 (7)	
H5	0.2044	0.4713	0.4374	0.096*	
C6	0.3477 (3)	0.31235 (19)	0.51256 (18)	0.0724 (6)	
H6	0.3376	0.2747	0.4541	0.087*	
C7	0.4408 (3)	0.25085 (15)	0.60686 (15)	0.0516 (4)	
C8	0.6935 (3)	0.08886 (14)	0.58002 (13)	0.0499 (4)	
C9	0.7082 (2)	0.03836 (14)	0.95003 (14)	0.0432 (4)	
C10	0.7920 (2)	−0.04822 (14)	1.04687 (14)	0.0439 (4)	
C11	0.8139 (3)	−0.02222 (17)	1.14823 (16)	0.0581 (5)	
H11	0.7708	0.0548	1.1588	0.070*	
C12	0.8976 (3)	−0.1075 (2)	1.23359 (17)	0.0704 (6)	
H12	0.9132	−0.0886	1.3023	0.084*	
C13	0.9583 (3)	−0.2187 (2)	1.22008 (19)	0.0710 (6)	
H13	1.0160	−0.2770	1.2792	0.085*	
C14	0.9358 (3)	−0.24622 (18)	1.1207 (2)	0.0716 (6)	
H14	0.9773	−0.3237	1.1113	0.086*	
C15	0.8529 (3)	−0.16125 (16)	1.03435 (16)	0.0577 (5)	
H15	0.8377	−0.1808	0.9659	0.069*	
C16	0.6757 (3)	−0.10936 (15)	0.68221 (15)	0.0526 (4)	
C17	0.8076 (3)	−0.21596 (14)	0.73653 (14)	0.0489 (4)	
C18	0.9977 (3)	−0.21949 (15)	0.75364 (15)	0.0553 (5)	
H18	1.0507	−0.1534	0.7249	0.066*	
C19	1.1108 (3)	−0.31812 (19)	0.81204 (17)	0.0675 (5)	
H19	1.2411	−0.3196	0.8238	0.081*	
C20	1.0355 (4)	−0.41383 (19)	0.85309 (19)	0.0795 (7)	
H20	1.1136	−0.4819	0.8931	0.095*	
C21	0.8486 (4)	−0.41166 (18)	0.8367 (2)	0.0851 (7)	
H21	0.7973	−0.4784	0.8656	0.102*	
C22	0.7330 (3)	−0.31380 (17)	0.77883 (18)	0.0671 (5)	
H22	0.6028	−0.3132	0.7679	0.080*	

### Atomic displacement parameters (Å^2^)

**Table:**

	*U*^11^	*U*^22^	*U*^33^	*U*^12^	*U*^13^	*U*^23^
S1	0.0863 (4)	0.0478 (3)	0.0852 (4)	0.0036 (3)	−0.0164 (3)	−0.0301 (3)
S2	0.0795 (4)	0.0420 (3)	0.0619 (3)	0.0045 (2)	0.0121 (3)	0.0005 (2)
O6	0.0759 (9)	0.0422 (7)	0.0499 (7)	0.0008 (6)	−0.0060 (6)	−0.0115 (6)
O7	0.0617 (9)	0.0473 (8)	0.0933 (11)	−0.0070 (7)	−0.0039 (7)	−0.0139 (7)
N1	0.0511 (8)	0.0349 (7)	0.0515 (9)	0.0007 (6)	0.0017 (6)	−0.0090 (6)
N2	0.0600 (9)	0.0381 (8)	0.0557 (9)	−0.0012 (7)	−0.0002 (7)	−0.0074 (7)
N3	0.0593 (9)	0.0430 (8)	0.0502 (9)	0.0006 (7)	−0.0080 (7)	−0.0139 (7)
N4	0.0681 (10)	0.0359 (8)	0.0503 (9)	0.0028 (7)	0.0040 (7)	−0.0085 (7)
C1	0.0435 (9)	0.0428 (10)	0.0598 (11)	−0.0055 (7)	0.0006 (8)	−0.0136 (8)
C2	0.0430 (9)	0.0369 (9)	0.0525 (10)	−0.0003 (7)	0.0050 (8)	−0.0031 (8)
C3	0.0693 (13)	0.0397 (10)	0.0671 (13)	0.0032 (9)	0.0049 (10)	−0.0062 (9)
C4	0.0797 (15)	0.0424 (11)	0.0831 (16)	0.0110 (10)	0.0013 (12)	0.0008 (11)
C5	0.0891 (16)	0.0591 (13)	0.0713 (15)	0.0162 (12)	−0.0161 (12)	0.0041 (11)
C6	0.0861 (15)	0.0581 (13)	0.0622 (13)	0.0073 (11)	−0.0157 (11)	−0.0064 (10)
C7	0.0535 (10)	0.0379 (9)	0.0553 (11)	0.0037 (8)	0.0004 (8)	−0.0049 (8)
C8	0.0668 (12)	0.0384 (9)	0.0400 (9)	0.0022 (8)	−0.0079 (8)	−0.0088 (7)
C9	0.0383 (9)	0.0412 (9)	0.0501 (10)	−0.0093 (7)	0.0036 (7)	−0.0091 (8)
C10	0.0332 (8)	0.0460 (10)	0.0506 (10)	−0.0102 (7)	0.0046 (7)	−0.0043 (8)
C11	0.0636 (12)	0.0558 (12)	0.0549 (11)	−0.0167 (10)	−0.0032 (9)	−0.0061 (9)
C12	0.0716 (14)	0.0806 (16)	0.0575 (12)	−0.0253 (12)	−0.0119 (10)	0.0019 (11)
C13	0.0523 (12)	0.0764 (16)	0.0692 (15)	−0.0135 (11)	−0.0067 (10)	0.0209 (12)
C14	0.0662 (13)	0.0516 (12)	0.0828 (16)	0.0014 (10)	0.0049 (11)	0.0031 (11)
C15	0.0589 (12)	0.0489 (11)	0.0595 (12)	−0.0048 (9)	0.0052 (9)	−0.0053 (9)
C16	0.0672 (13)	0.0398 (10)	0.0523 (11)	−0.0073 (9)	−0.0018 (9)	−0.0159 (8)
C17	0.0652 (12)	0.0359 (9)	0.0435 (9)	−0.0045 (8)	0.0058 (8)	−0.0108 (7)
C18	0.0713 (13)	0.0439 (10)	0.0480 (10)	−0.0079 (9)	0.0011 (9)	−0.0078 (8)
C19	0.0661 (13)	0.0658 (13)	0.0600 (12)	0.0041 (11)	0.0022 (10)	−0.0071 (10)
C20	0.0836 (17)	0.0568 (13)	0.0730 (15)	0.0153 (12)	0.0142 (12)	0.0095 (11)
C21	0.0904 (18)	0.0458 (12)	0.1012 (18)	−0.0055 (12)	0.0261 (14)	0.0099 (12)
C22	0.0682 (13)	0.0479 (11)	0.0770 (14)	−0.0055 (10)	0.0124 (11)	−0.0044 (10)

### Geometric parameters (Å, °)

**Table:**

S1—C1	1.6574 (18)	C6—H6	0.9500
S2—C8	1.660 (2)	C9—C10	1.482 (2)
O6—C9	1.222 (2)	C10—C15	1.381 (3)
O7—C16	1.219 (2)	C10—C11	1.390 (3)
N1—C1	1.335 (2)	C11—C12	1.379 (3)
N1—C2	1.409 (2)	C11—H11	0.9500
N1—HN1	0.8800	C12—C13	1.364 (3)
N2—C8	1.336 (2)	C12—H12	0.9500
N2—C7	1.435 (2)	C13—C14	1.375 (3)
N2—HN2	0.8800	C13—H13	0.9500
N3—C9	1.373 (2)	C14—C15	1.384 (3)
N3—C1	1.400 (2)	C14—H14	0.9500
N3—HN3	0.8800	C15—H15	0.9500
N4—C16	1.384 (2)	C16—C17	1.484 (3)
N4—C8	1.385 (2)	C17—C18	1.383 (3)
N4—HN4	0.8800	C17—C22	1.390 (3)
C2—C3	1.390 (3)	C18—C19	1.379 (3)
C2—C7	1.395 (3)	C18—H18	0.9500
C3—C4	1.376 (3)	C19—C20	1.368 (3)
C3—H3	0.9500	C19—H19	0.9500
C4—C5	1.368 (3)	C20—C21	1.361 (3)
C4—H4	0.9500	C20—H20	0.9500
C5—C6	1.382 (3)	C21—C22	1.378 (3)
C5—H5	0.9500	C21—H21	0.9500
C6—C7	1.375 (3)	C22—H22	0.9500
			
C1—N1—C2	132.18 (15)	C15—C10—C11	118.55 (18)
C1—N1—HN1	113.9	C15—C10—C9	117.42 (16)
C2—N1—HN1	113.9	C11—C10—C9	124.03 (16)
C8—N2—C7	123.32 (15)	C12—C11—C10	120.43 (19)
C8—N2—HN2	118.3	C12—C11—H11	119.8
C7—N2—HN2	118.3	C10—C11—H11	119.8
C9—N3—C1	130.47 (15)	C13—C12—C11	120.5 (2)
C9—N3—HN3	114.8	C13—C12—H12	119.7
C1—N3—HN3	114.8	C11—C12—H12	119.7
C16—N4—C8	127.91 (16)	C12—C13—C14	119.8 (2)
C16—N4—HN4	116.0	C12—C13—H13	120.1
C8—N4—HN4	116.0	C14—C13—H13	120.1
N1—C1—N3	113.59 (14)	C13—C14—C15	120.1 (2)
N1—C1—S1	129.80 (15)	C13—C14—H14	119.9
N3—C1—S1	116.61 (13)	C15—C14—H14	119.9
C3—C2—C7	118.45 (17)	C10—C15—C14	120.55 (19)
C3—C2—N1	125.64 (17)	C10—C15—H15	119.7
C7—C2—N1	115.87 (15)	C14—C15—H15	119.7
C4—C3—C2	120.1 (2)	O7—C16—N4	121.91 (18)
C4—C3—H3	119.9	O7—C16—C17	122.55 (18)
C2—C3—H3	119.9	N4—C16—C17	115.53 (17)
C5—C4—C3	121.14 (19)	C18—C17—C22	118.80 (18)
C5—C4—H4	119.4	C18—C17—C16	122.79 (16)
C3—C4—H4	119.4	C22—C17—C16	118.25 (17)
C4—C5—C6	119.4 (2)	C19—C18—C17	120.57 (18)
C4—C5—H5	120.3	C19—C18—H18	119.7
C6—C5—H5	120.3	C17—C18—H18	119.7
C7—C6—C5	120.3 (2)	C20—C19—C18	120.0 (2)
C7—C6—H6	119.9	C20—C19—H19	120.0
C5—C6—H6	119.9	C18—C19—H19	120.0
C6—C7—C2	120.62 (17)	C21—C20—C19	120.1 (2)
C6—C7—N2	120.04 (18)	C21—C20—H20	120.0
C2—C7—N2	119.32 (16)	C19—C20—H20	120.0
N2—C8—N4	116.00 (17)	C20—C21—C22	120.8 (2)
N2—C8—S2	124.29 (13)	C20—C21—H21	119.6
N4—C8—S2	119.66 (14)	C22—C21—H21	119.6
O6—C9—N3	121.30 (16)	C21—C22—C17	119.7 (2)
O6—C9—C10	121.83 (15)	C21—C22—H22	120.1
N3—C9—C10	116.87 (15)	C17—C22—H22	120.1
			
C2—N1—C1—N3	173.63 (15)	N3—C9—C10—C15	179.91 (15)
C2—N1—C1—S1	−6.5 (3)	O6—C9—C10—C11	179.29 (16)
C9—N3—C1—N1	1.1 (3)	N3—C9—C10—C11	−0.6 (2)
C9—N3—C1—S1	−178.77 (14)	C15—C10—C11—C12	1.1 (3)
C1—N1—C2—C3	4.2 (3)	C9—C10—C11—C12	−178.39 (16)
C1—N1—C2—C7	−173.60 (17)	C10—C11—C12—C13	−0.7 (3)
C7—C2—C3—C4	1.0 (3)	C11—C12—C13—C14	0.0 (3)
N1—C2—C3—C4	−176.74 (18)	C12—C13—C14—C15	0.4 (3)
C2—C3—C4—C5	−0.1 (3)	C11—C10—C15—C14	−0.7 (3)
C3—C4—C5—C6	−0.4 (4)	C9—C10—C15—C14	178.77 (16)
C4—C5—C6—C7	0.0 (4)	C13—C14—C15—C10	0.0 (3)
C5—C6—C7—C2	0.9 (3)	C8—N4—C16—O7	24.1 (3)
C5—C6—C7—N2	179.2 (2)	C8—N4—C16—C17	−154.85 (16)
C3—C2—C7—C6	−1.4 (3)	O7—C16—C17—C18	−153.22 (18)
N1—C2—C7—C6	176.58 (18)	N4—C16—C17—C18	25.7 (2)
C3—C2—C7—N2	−179.69 (16)	O7—C16—C17—C22	22.0 (3)
N1—C2—C7—N2	−1.7 (2)	N4—C16—C17—C22	−159.06 (17)
C8—N2—C7—C6	85.3 (2)	C22—C17—C18—C19	−0.3 (3)
C8—N2—C7—C2	−96.4 (2)	C16—C17—C18—C19	174.92 (17)
C7—N2—C8—N4	170.75 (15)	C17—C18—C19—C20	0.4 (3)
C7—N2—C8—S2	−6.4 (2)	C18—C19—C20—C21	−0.3 (3)
C16—N4—C8—N2	−5.1 (3)	C19—C20—C21—C22	0.1 (4)
C16—N4—C8—S2	172.23 (14)	C20—C21—C22—C17	0.0 (4)
C1—N3—C9—O6	−1.2 (3)	C18—C17—C22—C21	0.1 (3)
C1—N3—C9—C10	178.76 (15)	C16—C17—C22—C21	−175.32 (19)
O6—C9—C10—C15	−0.2 (2)		

### Hydrogen-bond geometry (Å, °)

**Table:**

*D*—H···*A*	*D*—H	H···*A*	*D*···*A*	*D*—H···*A*
N1—HN1···O6	0.88	1.88	2.633 (2)	141.8
N2—HN2···O7	0.88	1.99	2.680 (2)	133.9
N4—HN4···S2^i^	0.88	2.61	3.478 (2)	169.9

Symmetry codes: (i) −*x*+2, −*y*, −*z*+1.
